# Impact of Nucleic Acid Sequencing on Viroid Biology

**DOI:** 10.3390/ijms21155532

**Published:** 2020-08-01

**Authors:** Charith Raj Adkar-Purushothama, Jean-Pierre Perreault

**Affiliations:** RNA Group/Groupe ARN, Département de Biochimie, Faculté de médecine des sciences de la santé, Pavillon de Recherche Appliquée au Cancer, Université de Sherbrooke, 3201 rue Jean Mignault, Sherbrooke, QC J1E 4K8, Canada

**Keywords:** DNA sequencing, viroids, non-coding RNA, next-generation sequencing, third generation sequencing

## Abstract

The early 1970s marked two breakthroughs in the field of biology: (i) The development of nucleotide sequencing technology; and, (ii) the discovery of the viroids. The first DNA sequences were obtained by two-dimensional chromatography which was later replaced by sequencing using electrophoresis technique. The subsequent development of fluorescence-based sequencing method which made DNA sequencing not only easier, but many orders of magnitude faster. The knowledge of DNA sequences has become an indispensable tool for both basic and applied research. It has shed light biology of viroids, the highly structured, circular, single-stranded non-coding RNA molecules that infect numerous economically important plants. Our understanding of viroid molecular biology and biochemistry has been intimately associated with the evolution of nucleic acid sequencing technologies. With the development of the next-generation sequence method, viroid research exponentially progressed, notably in the areas of the molecular mechanisms of viroids and viroid diseases, viroid pathogenesis, viroid quasi-species, viroid adaptability, and viroid–host interactions, to name a few examples. In this review, the progress in the understanding of viroid biology in conjunction with the improvements in nucleotide sequencing technology is summarized. The future of viroid research with respect to the use of third-generation sequencing technology is also briefly envisaged.

## 1. Introduction

Since the discovery of viroids 53 years ago [1], they have remained one of the most fascinating biological molecules as they are the smallest known infectious RNA molecules (246 to 434 nucleotides (nt)) and are capable of autonomous self-replication without coding for any peptides. As a distinct class of pathogens, they are clearly distinguished from viruses. Although viroids demonstrate some structural and biological similarities with the hepatitis delta virus, the latter is about five times larger and encodes two proteins from a unique open reading frame [2,3]. Studies on viroids led to the discovery of some of the most interesting features in RNA biology, things such as the fact that non-translatable RNA can cause disease, that viroids are very small in size and possess a circular genome, and that they possess self-cleavage structures [4]. Research on viroids resulted in the development of several innovative methods such as nucleic acid purification and gel electrophoresis that became standard protocols that were subsequently used to confirm that prions are indeed infectious proteins that are devoid of any nucleic acids [5].

Although the potato spindle tuber disease, a degenerative disease of Irish potatoes, was reported in North America in 1922 [6], it took more than 40 years to identify and characterize the causative agent. More precisely, the research findings of Theodor Diener and William Raymer showed that the causative agent was protein-free RNA molecule 50–80 times smaller than the smallest known viral genomes. This agent, the first-ever reported “viroid” (term coined in the year 1971), was eventually named the potato spindle tuber viroid (PSTVd) [1,7]. The subsequent discoveries of the citrus exocortis viroid (CEVd), the causative agent of the citrus exocortis disease of citrus [8,9], and of the chrysanthemum stunt viroid (CSVd) that causes the stunting of chrysanthemum [10,11] further supported the existence of a new group of plant pathogens, specifically the viroids. Since then, several viroid species have been discovered in different host plants.

Viroid research grew exponentially as DNA sequencing technology evolved. The introduction of DNA sequencing by chemical approaches such as two dimensional chromatography [12] and both Maxam and Gilbert’s [13] and Sanger’s techniques [14] allowed researchers using these technologies, along with biochemical studies, to routinely sequence viroid RNAs. The exponential evolution in genomics driven by the development of the polymerase chain reaction (PCR), high-quality enzymes and the development of fluorescent automated DNA sequencing technologies rapidly contributed to the understanding of viroid biology. This led to the detection of several thousand viroid sequence variants that infect several plant species. These sequences are available in public databanks such as the National Centre for Biotechnology Information (NCBI). In 2006, the emergence of high-throughput sequencing techniques permitted the examination of billions of DNA and RNA templates [15]. Since then, these next-generation sequencing techniques have remained the most popular way of describing viroid quasi-species, viroid pathogenicity and with which to develop viroid resistant strategies.

This review focuses on the advancements in several areas of viroid biology as new sequencing technologies are introduced, with the caveat that the depth of the field makes it impossible to be comprehensive. The rapid evolution of biological tools, techniques and instruments has led to constant changes in sequencing technology, changes which are further helped by the ever-evolving analysis algorithms being developed. All of this creates substantial challenges as well as discoveries in viroid research. When examined in chronological order, as outlined in Table 1, the development of ever more powerful sequencing techniques resulted in major progress in the viroid research and massively contributed to our understanding of viroids. In other words, the evolution of sequencing approaches has been a driver of the study of viroids.

## 2. RNA Sequencing in the Characterization of Viroids

Although Friedrich Miescher discovered and isolated deoxyribonucleic acid (DNA) in 1869 [16], the field remained under-studied for almost a century because at that time proteins were thought to hold the genetic blueprint. However, the experiments performed by Oswald Avery, Colin MacLeod, and Maclyn McCarty in 1944 clearly demonstrated that DNA was capable of transforming the properties of cells [17]. In 1953, the works of James Watson, Francis Crick, Rosalind Franklin, and Maurice Wilkins contributed to the development of a conceptual framework for both DNA replication and for the encoding of proteins in nucleic acids [18,19]. In 1965, Robert Holley and colleagues were able to sequence the alanine transfer RNA (tRNA) from *Saccharomyces cerevisiae* by combining the techniques of ribonucleases (RNase) that were able to cut RNA chains at specific sites, analytical chemistry, and selective RNase treatments that produced both fully and partially degraded RNA fragments [20,21,22]. At the same time, the two-dimensional chromatographic fractionation technique for the detection of radiolabelled partially digested fragments was developed by Frederick Sanger and colleagues. This latter technique greatly facilitated the researchers ability to sequence short RNA molecules such as ribosomal and tRNA [12,23,24,25]. A protocol for the filling in of the ends of DNA molecules using DNA polymerase, while supplying each radioactive nucleotide individually and then measuring their rate of incorporation so as to be able to deduce the nucleotide sequence became available at the end of the 1960s [26,27].

### 2.1. Physical Characterization of Viroids

The discovery of a free nucleic acid property of the causal agent of potato spindle disease in the late 1960 [1] opened up research into one of the most interesting groups of non-coding RNA parasites, specifically the viroids. Potato spindle tuber disease infected plants exhibited elongated tubers in Irish potatoes in North America [6]. In the early 1960s, William Raymer and Muriel O’Brien conducted the first bioassay by transferring infectious agent from potato to tomato. This breakthrough finding permitted the visualisation of disease symptoms in tomato plants within 2 weeks of the treatment, which was exponentially faster than what is seen with potatoes where it takes a couple of years for the spindle tuber symptom to appear. In 1967, Theodor Diener, together with William Raymer, demonstrated that the pathogen was a free RNA molecule by a combination of density gradient centrifugation and enzymatic assays [1,28]. The early 1970s added two more species to this class of molecular pathogens, namely CEVd and CSVd, and eluded to the wide distribution of viroid hosts, which ranged from both perennial to annual plants and from flowering plants to fruit plants [8,9,10,11]. In the early years of viroid discovery, research was focused on understanding the physical and chemical properties of the viroid, the viroid’s replication, the host’s response to viroid infection and the primary sequence of viroid.

In 1971, two independent studies aimed at characterizing viroid RNA estimated that the molecular weights of PSTVd and CEVd were around 50,000–60,000 to 110,000–125,000 daltons based on the relative mobilities of their RNAs when electrophoresed in polyacrylamide gels of different concentrations [7,29]. The effect of the gel’s porosity on the relative migrations of PSTVd and CEVd demonstrated that they possessed properties characteristic of both double-stranded (ds) and single-stranded (ss) molecules. Specifically, by 1973, CEVd was eluted from methylated albumin and CF-11 cellulose as a double stranded RNA (dsRNA) molecule and was found to be susceptible to both RNases and formaldehyde inactivation, but to be resistant to inactivation by diethyl-pyrocarbonate (DEPC). In other words, it was demonstrating properties characteristic of both double stranded and single stranded RNA molecules [9,30,31]. Meanwhile, the fact that the viroid RNA was also partially resistant to phosphodiesterases suggested the possibility that it was circular in nature [28,31]. Further analysis of the 5′ and 3′ ends of the cucumber pale fruit viroid (CPFVd) indicated the absence of free termini, suggesting a covalently closed circular molecule [32]. In 1977, electron microscopy studies on purified PSTVd concluded that the viroid is indeed a single-stranded circular RNA [33]. In the same year, along with the circular forms, the presence of linear forms of the same viroid molecule were demonstrated, and both were found to be infectious [34]. High specific infectivity and resistance to thermal inactivation of even partially purified preparations were used for the prediction of the structural stabilities of the viroid RNA molecules [35]. The thermal denaturation profiles and NMR spectroscopy data obtained for PSTVd, CEVd, and CPFVd indicated the presence of highly based-paired structures that were rich in G-C bonds [30,33,36].

### 2.2. Sequence of the First Viroid RNAs (1978)

The knowledge of RNases, analytical chemistry and radiolabelling two-dimension (2D) fractionation techniques was applied to the characterization of viroid RNA. The two-dimensional fingerprints of RNase T_1_ digests, coupled with pancreatic RNase digests of both ^125^I- and ^32^P-labelled viroid RNAs, revealed the nucleotide sequences of both PSTVd and CEVd [37,38]. The fingerprint patterns of both viroids remained constant, regardless of the host species that had been infected [39]. This discovery was highly significant in the understanding viroid biology as, previously to this, it was virtually impossible to separate PSTVd and CEVd based on either biological tests or infection assays [35]. In 1978, using 5′-end ^32^P-labelled RNAse T1 and A fragments, the primary sequence of the 359-nt long PSTVd was elucidated [40]. This breakthrough led to conclusive evidence on the physical size and structure of a viroid RNA, and even accommodated a previously proposed model of a covalently closed, circular RNA with a high degree of internal base-pairing. The elucidation of the viroid sequence confirmed several features of this fascinating new class of molecular pathogens, but little or no information could be deciphered about the replication, the pathogenesis and the host–pathogen interactions of the viroid. Additionally, the absence of an AUG triplet in the primary sequence, as well as in its complementary sequence, led to the conclusion that neither the primary sequence nor its putative complementary sequence can function as a messenger RNA [35]. That said, the determination of the sequence of PSTVd provided a strong foundation for viroid research by permitting the conclusion that it is a regulatory RNA pathogen that was clearly distinct from viruses.

## 3. Early Sequencing Methods (1980s)

**Figure 1:**
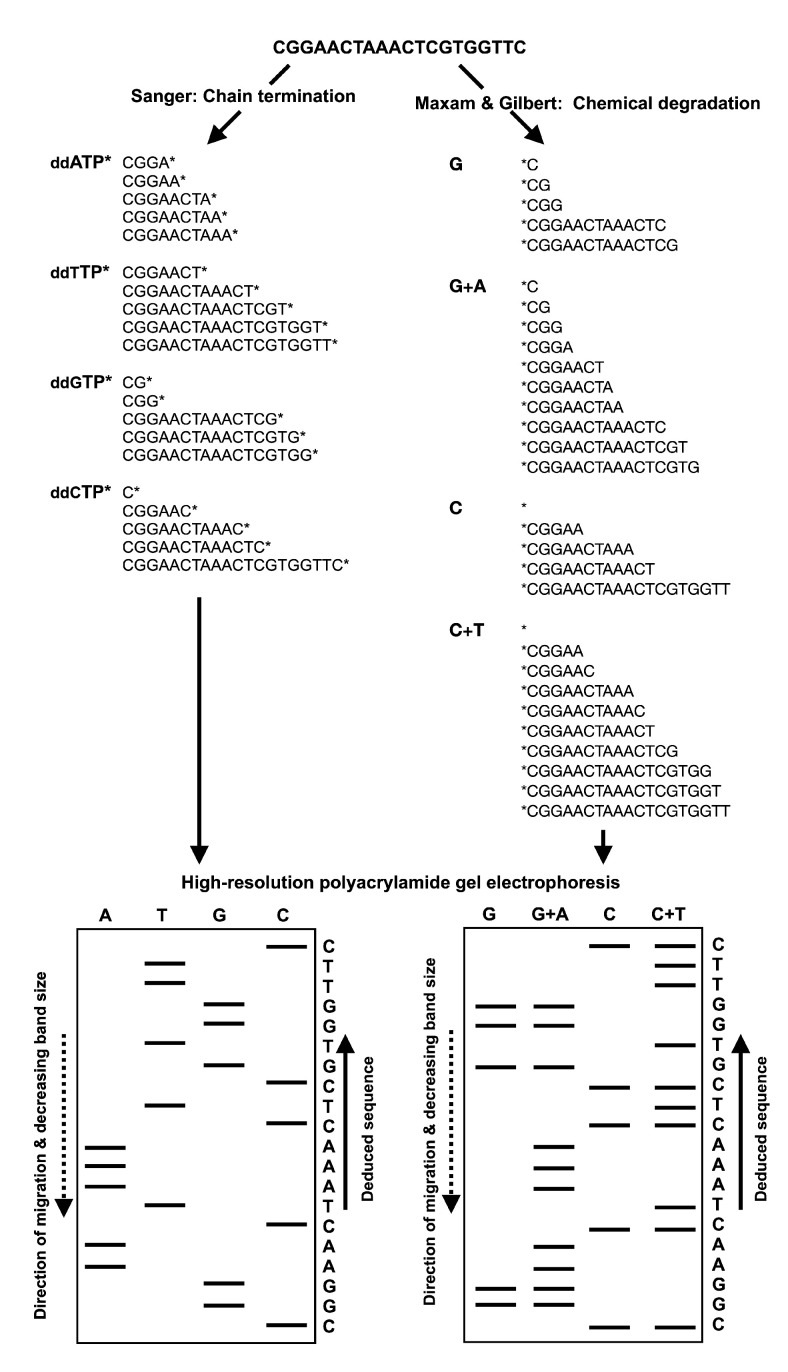
Schematic illustrations of Sanger’s chain-termination and Maxam and Gilbert’s chemical sequencing techniques. In Sanger’s sequencing method, radiolabeled ddNTP nucleotides of a specific type (i.e., ddATP, ddTTP, ddGTP and ddCTP) are included in the DNA polymerase reactions at low concentrations along with the dNTPs. In each of the four reactions, polymerization will continue to extend with dNTPs until a ddNTP is incorporated, generating DNA strands of varying lengths. The DNA fragments are then visualized by high-resolution polyacrylamide gel electrophoresis. The nucleotide sequence is then deduced by finding the lane in which the band is present for a given site, as the 3′ terminating labeled ddNTP corresponds to the base at that position. Maxam and Gilbert’s method requires the radiolabeling (^32^P) of the 5′ phosphate moiety of the DNA fragment to be sequenced prior to chemical treatment for the selective removal of the base from a small proportion of the DNA molecules. Guanine is methylated by dimethyl sulfate, formic acid depurinates the purines (adenine and guanine); hydrazine hydrolyzes the pyrimidines (cytosine and thymine) and hydrazine in the presence of high salt (sodium chloride) concentrations can only react with cytosine. Piperidine is then used to cleave the phosphodiester backbone at the position of the modified base, yielding fragments of various lengths. The DNA fragments are then separated by high-resolution polyacrylamide gel electrophoresis in order to deduce the nucleotide sequence. The guanine (G) bands are present in both the G and A+G (purine) lanes, while the adenosine (A) band is present only in A+G lane. Similarly, cytosine (C) is indicated by the presence of bands in both the C and C+T (pyrimidines) lanes, while thymidine (T) bands are present only in C+T lane.

In late 1960s, parallel to the development of the two-dimensional fractionation sequencing techniques, the technique of DNA sequencing by location-specific primer extension strategy using radioactive nucleotides in the presence of DNA polymerase and then measuring the incorporation rate in order to deduce the sequence was developed [26,27]. Since this technique used laborious analytical chemistry and fractionation procedures, the method was limited to short stretches of DNA. This drawback was rectified in 1977 by the replacement of the two-dimensional fractionation step with a single separation based on polynucleotide length via electrophoresis through polyacrylamide gels, either by Sanger’s “chain-termination” method, which was also known as the “dideoxy technique” or simply “Sanger sequencing” [41], or by Maxam and Gilbert’s “chemical degradation” method [13]. Figure 1 outlines both the dideoxy and the chemical degradation techniques. This might be considered the real birth of DNA sequencing, and it was a widely adopted as a sequencing technique due to its feasibility [42]. Meanwhile, in 1970, Howard Temin and David Baltimore independently isolated reverse transcriptases from both Murine leukemia virus and Rous sarcoma virus [43,44]. This facilitated the viroid RNA sequencing by reverse transcribing RNA into DNA, the template required by the early sequencing methods.

### Viroid Classification

In the 1980s, the complete nucleotide sequences of at least 10 viroids were determined by either 2D fractionation sequencing technology, or by generating cDNAs from the viroid RNA using reverse transcriptase and then sequencing them using the dideoxy method and oligonucleotide primers. All of these viroid particles had molecular weights in the 110,000 to 170,000 daltons range and had single-stranded RNAs of 246 to 371 nucleotides in length [45]. Comparison of the nucleotide sequences revealed that almost all of the viroids possessed a conserved central region, that was mostly composed of base pairs (bp) and which was 19 nucleotides in length [46,47], and a purine-rich tract located around positions 25 to 50 that was located to the left of this conserved region [45,48]. Additionally, two specific regions associated with specific functions were identified. One of these regions determined the variation in the pathogenicity of the PSTVd isolates [40], while the other was involved in the non-enzymic cleavage of an avocado sunblotch viroid (ASBVd) dimeric transcript between the residues located at positions 55 and 56 [49]. Examination of all of the sequence data showed that PSTVd, CSVd, CEVd, tomato planta macho viroid (TPMVd) and tomato apical stunt viroid (TASVd) exhibited 60%–80% homology with each other. Hop stunt viroid (HSVd), CPFVd, grapevine viroid (GV) and coconut cadang-cadang viroid (CCCV) formed a different clade that possessed a central conserved region (CCR), but that were only distantly related to the PSTVd group. However, these two clades differ distinctly from ASBVd which lacked CCR [45,50]. Based on the sequence homologies among the seven viroid species, functional domains of these viroids with five structurally distinguishable regions were proposed: A conserved central region (CCR) capable of forming two alternative structures, a region that determined the pathogenicity (P), a region that exhibited high sequence variability (V) and two terminal domains that were interchangeable between viroids (the left terminal [LTR] and the right terminal [RTR] regions) [49]. ASBVd was excluded from this model since its sequence was significantly different from all other known viroids.

**Figure 2:**
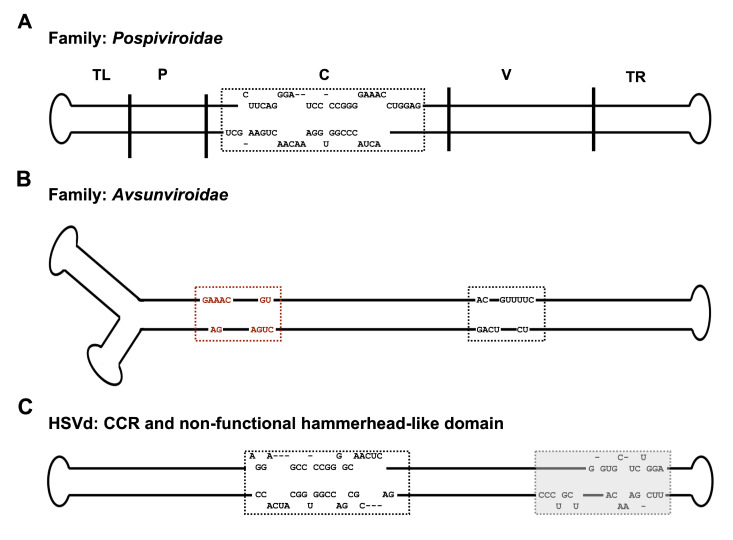
Primary and secondary structural features used for viroid classification. Schematic representations of (**A**) The rod-like secondary structure of potato spindle tuber viroid (PSTVd). The five functional domains are shown on the secondary structure of PSTVd: The Terminal left (TL), Pathogenicity (P), Central (C), Variable (V), and Terminal right (TR) domains are delimited by the vertical solid lines and are named accordingly. The sequence of the central conserved region (CCR), which is the characteristic feature of the members of the family *Pospiviroidae*, is indicated within the box. (**B**) The branched secondary structure of avocado sunblotch viroid (ASBVd). The conserved nucleotides of the hammerhead’s catalytic core, the characteristic feature of the members of the family *Avsunviroidae*, are boxed. The sequences within the red and black boxes denote the hammerhead self-cleaving motifs formed in the viroid’s (+) and (−) strands, respectively. (**C**) The rod-like secondary structure of hop stunt viroid (HSVd) showing both the CCR (black color boxed) and a non-functional hammerhead-like domain (shadowed box), respectively.

The detection of new viroid species, coupled with the accumulation of viroid sequence data, led to the determination of two alternate features, the CCR domain and a hammerhead structure, that were extremely well conserved among the different isolates of a viroid and which played a crucial role in the viroid’s replication [51,52,53,54,55]. Specifically, those viroids lacking a CCR domain possessed a hammerhead self-cleavage ability which served as the major criteria for classifying viroids in two families [56,57]. The members of the family *Pospiviroidae*, such as PSTVd, are characterized by having a CCR, but lacking hammerhead self-cleavage. In contrast, the members of family *Avsunviroidae*, like ASBVd, showed hammerhead self-cleavage, but lacked a CCR domain. That said, the molecular variability comparison of HSVd revealed the presence of a CCR as well as that of a non-functional hammerhead-like domain, the distinctive features of the members of both the families, suggesting that HSVd is an evolutionary link between the *Pospiviroidae* and *Avsunviroidae* [58] (Figure 2).

## 4. Automated DNA Sequencing

### 4.1. Dideoxy Chain-Termination Sequencing (1990–To Date)

During the subsequent years, due to its robustness and ease of use, the Sanger dideoxy chain-termination sequencing method rapidly improved in its capacity, capability and applications. Among the improvements responsible for this growth in the usefulness of Sanger sequencing perhaps the most important included the replacement of radiolabelling by fluorescence-based detection and the separation of short DNA fragments via capillary-based electrophoresis, all of which increased the length of the nucleotide sequences that were elucidated [59,60,61,62]. Together, all of the improvements contributed to the development of a semi-automated version of the original Sanger strategy. Subsequent developments in molecular biology techniques, such as the recombinant DNA technologies, discovery of the polymerase chain reaction (PCR) and the developments in the thermocycler [63,64,65], gave birth to automated DNA sequencing machines [66]. Further developments in DNA sequencing technology eventually paved the way to commercial DNA sequencing machines. These first-generation automated DNA sequencing machines are capable of reading approximately 1000 bases with an achievable accuracy of 99.99% by capillary electrophoresis [67]. Although these first-generation DNA sequencing machines were widely used in research, they presented significant hurdles in their use in the study of larger genomes due to their high operational costs, laborious preparation steps required and low throughput results. However, a single run of these sequencing machines was able to provide the complete genome sequence of a viroid. Consequently, this technology is still routinely used to characterize viroid genomes today.

### 4.2. Next-Generation Sequencing (2005–To Date)

**Figure 3:**
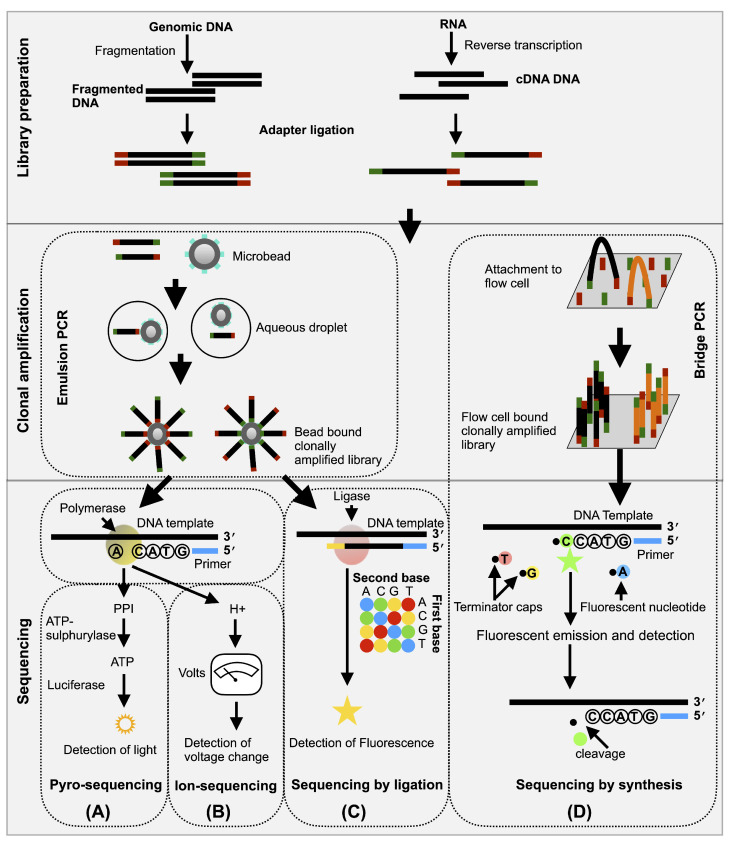
Overview of next-generation sequencing. Clonally amplified template DNA bound to beads are sequenced by either pyrosequencing (**A**), ion-sequencing (**B**) or ligation (**C**), while clonally amplified templates bound to glass flow cells are sequenced by the synthesis technique. All of the NGS techniques include three main steps: (i) Library preparation; (ii) clonal amplification; and, (iii) sequencing. The library to be sequenced is prepared either by fragmenting genomic DNA, or by reverse transcribing the RNA into cDNA (hereafter referred to as DNA). An adapter sequence is added to either side of the DNA which permits the hybridization of the library to the sequencing chips and provides a universal primer binding site for the sequencing primers. In (**A**), (**B**), and (**C**), the library fragment is amplified on solid surface beads by emulsion PCR using covalently attached DNA linkers that hybridize to the library adapters. The sequence of each cluster is optically read either through the generation of light during the polymerase reaction (as in **A**), or change in the voltage due to the release of H ion during the addition of a nucleotide (as in **B**) or by fluorescent signal during ligation (as in **C**), from repeated cycles of nucleotide incorporation. In (**D**), a library fragment is bound to an optically transparent glass flow cell with covalently attached DNA linkers that hybridize the library adapters by bridge PCR. The chip bound clusters’ sequences can be acquired by the detection of the fluorescence of the reversible-terminator nucleotides at the end of the proceeding extension reaction.

In the mid-1990s, concurrent with the development of large-scale sequencing methods that were based on Sanger sequencing, new DNA sequencing techniques called Next-Generation Sequencing (NGS or high-throughput sequencing or deep sequencing) or second-generation technology were developed. In contrast to the previous sequencing techniques, NGS were capable of: (i) Preparing a DNA fragment library without cellular cloning; (ii) being highly scalable; and, (iii) enabling DNA fragments to be multiplexed. Consequently, NGS received significant attention from biologists hoping to achieve whole-genome projects in a single run. Briefly, NGS is accomplished by fragmentation of entire genome into small pieces so as to prepare a library. This is then followed by the random in vitro clonal amplification of these DNA fragments and, finally, by the sequencing of the immobilized DNA on a solid substrate using either: (i) pyro-sequencing on beads [68]; (ii) ion-sequencing (first “post-light sequencing” technology) [69]; (iii) ligation on beads (polony sequencing) [70]; or, (iv) synthesis on a glass substrate [71,72]. The principles underlying each of these technologies are shown in Figure 3. One of the drawbacks of second-generation technology is the use of PCR amplification during library preparation as this introduces a bias in reading distribution which affects the ultimate sequence result. NGS platforms, their capabilities, the principles behind each method and their advancements have been reviewed elsewhere [15,67].

### 4.3. Structure of Viroids

As viroids consist of naked RNAs that do not code for any proteins that could be helpful in their life cycles, the elucidation of the structures adopted by viroids was crucial for understanding not only the viroid–host interaction, but also a viroid’s infection cycle. Since the discovery of viroids their molecular structures have remained unknown as they lacked any of the physically recognizable characteristics of infectious preparations such as the cellular structure of bacteria or the coat protein of a virus. Direct biophysical and biochemical analysis on highly purified viroid molecules revealed that they consist of a single-stranded RNA molecule of about 360 nucleotides in length [32]. When the primary sequence of PSTVd was elucidated by the RNase method in 1978, the unique structural features of viroid were finally unraveled. However, the sequencing was severely delayed due to both the insufficient labeling of the viroid molecules and the low rate of viroid replication. In the end, the primary sequence was elucidated by a combination of the conventional and the rapid gel-sequencing techniques followed by overlapping sequencing [40]. By correctly arranging the resulting primary nucleotide sequence of PSTVd, a maximum number of intramolecular base pairs was obtained. This produced a largely double-stranded, rod-like structure [38]. The determination of viroid sequences was greatly enhanced by the introduction of automated sequencing technologies as a single sequencing run yielded a full length read of the viroid’s nucleotide sequence. The introduction of computer-based, thermodynamic folding facilitated the deduction of a viroid’s secondary structure from the obtained primary nucleotide sequences. The resulting structural features became one of the most important criteria for the classification of viroids [57].

Recent developments of certain biochemical techniques, specifically the selective 2′-hydroxyl acylation analyzed by primer extension (SHAPE) and genotyping, combined with computer algorithms permitted the determination of the secondary structures of all viroids in solution, as well as of a limited number *in vivo* [73,74,75]. However, the structures determined using these modern structural elucidation methods exhibited deviations from the classical rod-like structure for some of the members of the family *Pospiviroidae*, more precisely with both the columnea latent viroid (CLVd) and the citrus viroid OS (CVd-OS) [73,76]. Since these techniques helped to predict the secondary and tertiary structures of viroids as close as possible to their natural confirmations, the structural hallmarks for the identification of each genus were proposed [77]. The structure of viroids has been reviewed elsewhere [78].

### 4.4. Mutagenic Studies used to Understand Viroid Pathogenicity

Due to the size and non-coding nature of viroids, site-directed mutations were widely used to study the pleiotropic functions of the viroid RNA genome. By comparing the nucleotide sequences of naturally occurring isolates of PSTVd, isolates which differed in the severity of symptoms that they incite were discovered which eventually helped in identifying a region associated with pathogenicity [79]. However, site-directed mutagenic studies helped to reveal several exciting features of viroids. For instance, a single nucleotide change in the upper CCR abolished the infectivity of CEVd [80]. Similar observations were made for both PSTVd and HSVd [81,82]. Although early molecular cloning techniques permitted the construction of mutant infectious cDNA clones, the PCR based introduction of site-directed mutation, and its confirmation by automated sequencers, allowed the manipulations to be performed much more simply.

The mutagenic studies were extended to study not only the pathogenicity, but also the replication of viroids. For instance, the C259U mutation allows for the efficient replication of the KF440-2 isolate of PSTVd in tobacco (*Nicotiana tabacum*) [83]. On the other hand, the mutation U257A transformed PSTVd-Int (i.e., an intermediate strain of PSTVd) into a lethal strain when assayed in tomato [84]. Previously, it was found that changing the U257A mutation to either C or A in PSTVd-Int resulted in a five- to ten-fold increase in the replication rate in tobacco protoplasts [85]. Similarly, mutagenic studies helped in understanding the host-dependent mutations like those observed with the apple fruit crinkle viroid (AFCVd) [86]. Mutagenic studies on coleus blumei viroid 1 (CbVd-1) revealed that a point mutation at position 25 in loop five incites the potential to transmit the viroid through seeds [87]. Additionally, genome-wide mutations on PSTVd have been performed in order to understand the motifs critical for its replication and trafficking [88].

### 4.5. Whole Genome Sequencing on Viroid Isolates Leads to Quasi-Species

Viroids are very diverse in nature as each viroid species has sequence variants/isolates which are capable of infecting and inducing an array of symptoms in susceptible host plants. In addition, each isolate is capable of forming a genetic heterogeneity in infected host plants. As early as 1983, at least four CEVd isolates (CEV-DE25, CEV-DE26, CEV-A, and CEV-C) were identified [46]. Moreover, the sequencing of recombinant DNA clones prepared from purified CEV-A revealed the presence of at least one other CEVd sequence variants [46]. The sequencing of cDNAs prepared from two new purified CEVd Australian field isolates indicated that both isolates consisted of a mixture of sequence variants that were present in the CEVd in infected plants. Together these studies revealed the heterogeneity characterizing the viroid. A total of seventeen CEVd variants have been sequenced, and they form two classes of sequence variants that differ by a minimum of 26 nucleotides out of a total of 370 to 375 residues. Interestingly, upon infection, one class produced severe symptoms while the other induced mild symptoms in tomato plants [89]. This data revealed, for the first time, the presence of both viroid isolates and genetic heterogenicity.

During replication, the nuclear replicating *Pospiviroidae* members use the host’s DNA dependent RNA polymerase II, whereas the chloroplast replicating *Avsunviroidae* members recruit host’s nuclear-encoded polymerase (NEP) [90]. Since viroids force the host’s polymerase to use the viroid’s RNA instead of their natural host DNA as a template, the replication becomes error-prone as the relevant polymerases are unable to proofread [90]. Hence, during infection, every viroid variant is capable of producing its own population dynamics within the infected host plant, a concept called quasi-species that was first proposed in 1993 [91]. Similar to CEVd, the sequence variants of PSTVd were classified as being mild, intermediate, severe, or lethal based on the symptoms they induced in tomato plant cultivar Rutgers. By 1997, around forty different PSTVd sequence variants had been identified. Comparative analysis of these available PSTVd sequences revealed that they differ from each other by only a few nucleotides over a total RNA length of 359–360 nucleotides. Interestingly, most of these mutations were located in the P and V domains [92,93,94,95,96,97]. The development of first-generation sequencing machines helped with the routine sequencing of viroid species, and ultimately resulted in a large amount of sequence data for each viroid species. Comparison of the sequence data revealed the presence of viroid isolates, as well as of quasi-species, in other viroid species such as ASBVd [50,98], HSVd [99,100,101], CbVd-1 [102], grapevine yellow speckle viroid 1 (GYSVd-1) [103], citrus viroid III (CVd-III) [104], and peach latent mosaic viroid (PLMVd)) [53,105].

DNA sequencing by the first-generation sequencers permitted the study of the quasi-species nature of viroids by analysis of the multiple clones recovered from a single plant infected with a known viroid sequence. For example, the demonstration of the sequence variability of the PSTVd progeny recovered from tomato plants inoculated with cloned PSTVd cDNA validated the genetic heterogenicity of PSTVd [106]. On the other hand, the heat treatment of hop plants infected with hop latent viroid (HLVd) revealed a decrease in viroid titer, but an increase in HLVd sequence variability, thus indicating that the quasi-species phenomenon helps a viroid adapt to environmental changes [107]. Chrysanthemum plants inoculated with an *in vitro* transcript of chrysanthemum chlorotic mottle viroid (CChMVd), a member of the family *Avsunviroidae*, at the onset of symptoms, permitted the screening of the mutations found in the progeny and the determination of mutation rate for viroids. Taking into account the mutations present at the 15 core nucleotides, as well as of the 32 sites preceding the self-cleavage site, in two hammerhead regions of CChMVd revealed one mutation per 400 residues, the highest reported mutation rate for any given biological species [108].

Although the forced utilization of the host’s polymerase during the viroid’s replication plays a major role in the quasi-species phenomenon, the influence of the host’s selection pressure and of the viroid’s adaption cannot be ignored. Analysis of both accumulation and sequence diversity data for both the nuclear-replicating chrysanthemum stunt viroid (CSVd) and the chloroplast replicating CChMVd recovered from double infected chrysanthemum plants revealed the “survival of the flattest”. That said, under optimum conditions, the viroid species that accumulates faster and that possesses genetic homogeneity (i.e., CSVd) outcompeted the one with a slow accumulation and a high degree of diversity (i.e., CChMVd), indicating that an increased mutation rate negatively affects the survival of a slow accumulating viroid species. However, it should be noted that CChMVd out competed CSVd when the mutation rate was increased [109]. Additionally, the analysis of the progenies derived from different citrus host species inoculated with citrus dwarfing viroid (CDVd) suggested that genetic diversity is host species-dependent, irrespective of the time that the viroid has been in association with the host plant [110].

The development of a high-throughput sequencing platform capable of processing multiple DNA sequences in parallel (i.e., massively parallel sequencing, permitted the sequencing of millions of viroid RNA molecules in a single run and provided a greater insight into viroid genetic heterogenicity than did the first-generation sequencers. Therefore, recently, NGS has been used to study the genetic diversity of both *Avsunviroidae* and *Pospiviroidae* members. In order to understand the evolution of the sequence heterogeneity of PLMVd, a member of the *Avsunviroidae* family, a GF305-indicator peach tree was infected with a single sequence variant [110]. At six months post-inoculation, the circular RNAs were extracted and purified and were then sequenced using a 454 GS-FLX Titanium pyrosequencing platform. The data obtained included a total of 291,959 reads consisting of 3939 different PLMVd variants. Detailed analysis of the results revealed that, when the variant sequences were compared to the inoculated PLMVd sequence, 50% of the positions were found to be conserved. This included several small stretches and a small motif reminiscent of a GNRA tetraloop. The majority of the sequence variants recovered possessed an average of 4.6 to 6.4 mutations when compared to the inoculated PLMVd variant. In order to verify whether or not the high rate of mutation observed for a chloroplast replicating viroid (i.e., CChMVd) is a general feature of viroids, representative viroids from both families (specifically Eggplant latent viroid [ELVd] and PSTVd) obtained from a common host (eggplant) were sequenced on an Illumina MiSeq platform. The data revealed that the mutation rates ranged from 1/1000 to 1/800 for ELVd and from 1/7000 to 1/3800 for PSTVd depending on the particular sequencing run, indicating that the mutation rates of PSTVd, and potentially those of other nuclear viroids, appeared to be significantly lower than those of plastid replicating viroids, and to in fact be closer to those of some RNA viruses [111]. In contrast to this finding, analysis of the quasi-species of PSTVd by re-construction of the small RNAs (PSTVd-sRNAs) obtained from the deep-sequencing data of plants infected with different variants revealed the mean error rate per nucleotide position was less than 5000, a value close to that calculated for the members of *Avsunviroidae* (i.e., 2500) [112]. However, while comparing these two studies, readers should note that different techniques were used for preparing the viroid libraries in order to be able to analyze the quasi-species. More recently, in order to understand the shift in viroid population dynamics due to mutations over the course of infection, the ten most abundant sequence variants of PSTVd strain RG1 expressed at different time intervals in PSTVd infected tomato plants were identified by high-throughput sequencing [113]. The sequence variants forming a quasi-species were subjected to both the identification of the regions favoring mutations, and to the effects of the mutations on both the viroid’s secondary structure and on the viroid derived small RNAs (vd-sRNA). The data revealed that the “master” sequence (i.e., the sequence used for the infection) represented only 25% of the population at week 1 post PSTVd infection, and that it reached an equilibrium at 70% in week 2 that was maintained throughout the course of the infection. Some sequence variants were relatively abundant at week 1 post PSTVd infection and then decreased in abundance, while others increased.

### 4.6. Small RNA Sequencing and Viroid Induced RNA Silencing

RNA silencing is a natural antiviral defense mechanism in plants and animals against either double-stranded or highly structured RNA pathogens which results in both the production and the accumulation of invading RNA pathogen-specific 21- to 24-nt long small RNAs (sRNAs) [114]. In the late 1980s, comparisons of viroid nucleotide sequences with those of cellular RNAs revealed the presence of a number of sequence similarities. For instance, five viroids “species” that cause disease in tomato showed a high degree of sequence complementarity with a stretch of 36–53 nucleotides of the 7S RNA (SRP) of tomato. Furthermore, two domains of PSTVd and a portion of (−) strand of PSTVd showed complementarity with the mammalian U3B and UI snRNAs, respectively [115,116]. Although the concept of RNA silencing was yet not developed, these findings lead to the proposal of thermodynamically favored base-pairing between the host RNA and the viroid RNA *in vivo*. The actual observation of this phenomenon led to the hypothesis that viroids could interfere with the host’s RNA and, thus, induce disease symptoms [117,118].

The petunia plants (*Petunia hybrida*) possessing additional transgene copies of the chalcone synthase gene (CHS; i.e., a flower pigmentation gene), expressed an array of pigmentations, including intense purple, patterns of purple and white and completely white flowers, a phenomenon referred to as “co-suppression” [119,120]. Elsewhere, the agrobacterium-mediated sequential introduction of two T-DNA vectors encoding different selectable markers into a tobacco (*Nicotiana tabacum*) genome resulted in the inactivation in the double-transformant of the first T-DNA encoded selectable marker population following the introduction of the second vector [118]. In this work the authors reasoned that the second vector, which shared substantial sequence homology with the first vector, may have initiated the methylation of the latter. Importantly, this study provided additional insights into homology-dependent gene silencing mechanisms in plants. The first demonstration of RNA silencing of a viroid molecule was reported in a PSTVd over-expression system in the year 1994 [121].

Seven years later, vd-sRNA were detected in plants infected with viroids such as PSTVd, ASBVd, PLMVd, and CChMVd, suggesting that these viroids are the targets of post-transcriptional gene silencing (PTGS) irrespective of their subcellular localization during replication [122,123,124,125]. Experiments implied that vd-sRNA: (i) were produced from both the (+) and (−) strands of the viroid RNA; and, (ii) play a crucial role in the mediated viroid symptom expression [125,126]. In 2007, two groups cloned and sequenced PSTVd-sRNA purified from PSTVd infected plants [127,128]. Although the number of sequenced vd-sRNAs was limited, both studies revealed that both the left and the right terminal domains are hotspots of PSTVd-sRNA biogenesis. Furthermore, another study provided the first evidence that viroid infection triggers the host’s RNA silencing machinery to produce biologically active vd-sRNA [128]. In the same year, the CEVd-sRNA was sequenced using first-generation sequencers [129]. In contrast to PSTVd sRNA biogenesis, CEVd showed a high concentration of sRNA derived from the right terminal domain [129]. The availability of NGS platforms boosted the identification and characterization of 21- to 24-nt long sRNAs derived from viroids of both families [130,131,132,133,134,135,136,137,138]. The profiling of vd-sRNA on their respective viroid RNAs suggested that the genomic (+) strand produced more sRNA than did the antigenomic (−) strand. This could be due to the differential accumulation of the two polarities in viroid infected plants [55,138]. It also revealed the regions on the viroid molecule that are susceptible to the host’s RNA silencing machinery. Based on these data, a set of transgenic plants expressing vd-sRNAs derived from various functional domains of PSTVd were developed in order to verify the RNA interference (RNAi) mediated inhibition against PSTVd infection [139]. Interestingly, these transgenic lines showed a certain level of resistance to viroid infection.

The accumulation of large scale vd-sRNA data revealed that viroids are both the inducers and the targets of RNA silencing in viroid infected plants, suggesting that this vd-sRNA might target host mRNA and thus play a role in viroid pathogenicity [125,126,140]. In one study, the authors hypothesized that the miRNA-dependent (mis)regulation of transcription factors causes the viroid induced symptoms, as their study revealed sequence complementarity between a region of the pri-miR403 (the miRNA involved in leaf development via the regulation of transcription factors) of tomato (specifically the 30 nucleotides located downstream of the pre-miR403’s 3′ end) and PSTVd strain AS1 (specifically the reverse complement of positions 30 to 46) [141]. A variant of PLMVd, calico (PC) which contains a specific 12–14 nucleotide hairpin insertion, induces albinism in susceptible host plants [142]. Taking advantage of this sequence-dependent symptom induction, the authors determined that the two sRNA derived from this insertion of PLMVd-PC targeted the mRNA encoding the chloroplastic heat-shock protein 90 (cHSP90) for cleavage, thus implicating RNA silencing in the modulation of a host gene’s expression by a viroid [140]. Meanwhile, transgenic *Nicotiana tabacum* and *Nicotiana benthamiana* plants expressing the sequence corresponding to the virulence modulating region (VMR) of PSTVd that was predicted to target a soluble inorganic pyrophosphatase mRNA on an artificial miRNA backbone induced abnormal phenotypes that closely resembled what is seen in PSTVd infected plants [143]. The direct interaction between vd-sRNA and the predicted host target sequence was demonstrated by expressing the PSTVd-sRNA that was predicted to target the callose synthase gene in an artificial miRNA experiment in a transient expression system [144]. Based on both *in silico* and *in vivo* studies, several research groups have demonstrated the involvement of vd-sRNAs in the down regulation of host genes [145,146,147,148,149,150,151]. This was recently reviewed elsewhere [114]. Lastly, both degradome studies and the transcriptome analysis of RNA-sequencing (RNA-seq) data obtained from different viroid–host combinations revealed that viroid infection induces the genome-wide degradation of host RNA either by direct interaction (vd-sRNA), or by phased secondary small interfering RNAs (Phasi-RNA) [152,153,154,155]. Importantly, this progress would have not been possible without the NGS revolution.

### 4.7. RNA-Sequencing and the Effect of a Viroid on the Host’s Transcriptome

RNA-sequencing (RNA-seq) is widely used to study the effect of viroids on global transcription in the host plants. This is done by comparing the transcription data obtained from a control plant at a given moment, such as at a specific developmental stage or under a specific physiological condition, with that from an infected plant, as well as by comparing the data from normal and diseased tissues. Prior to RNA-seq, differential gene expression studies in viroid infected plants were performed using both macro- and microarrays [138,150,156,157,158,159,160]. Although these technologies required a priori knowledge of the sequence, as well as presenting technical concerns over issues such as cross-hybridization, poor quantification of both lower and highly expressed RNAs [161], the data obtained suggested that global transcriptomic alterations were triggered by viroid infection. Prior to NGS based RNA-seq technology, Expressed Sequence Tag (EST) libraries obtained from Sanger Sequencing were used in transcriptomics. However, it was not well applied in viroid biology. Recently, RNA-seq has been widely used in various viroid–host combinations for the analysis of the changes in gene expression as a means of generating a far more precise estimate of both the mRNA levels and of the transcript isoforms at a much lower cost as compared to microarrays technologies [162,163]. In summary, RNA-seq data revealed changes in the expression of genes involved in photosynthesis, cell wall structure, RNA regulation, the biosynthetic pathways of hormones, protein metabolism, and defense and stress responses in viroid infected plants.

To date, the majority of RNA-seq studies have been focused on the transcriptome analysis of plants infected with the members of the family *Pospiviroidae* [152,163,164,165]. RNA-seq data analysis of cucumber plants infected by two HSVd variants that differ in pathogenicity at different times post-inoculation revealed that HSVd infection depressed photosynthesis, disrupted phytohormone homeostasis, and also triggered both basal defense responses and the expression of genes coding for RNA-dependent RNA polymerase [163]. Hop leaves infected with HSVd revealed major differential expression in the genes involved in defense and in those involved in both lipid and terpenoid metabolism [164]. Genome-wide transcriptome analysis of hop plants infected with citrus bark cracking viroid (CBCVd) resulted in the massive modulation of the activity of over 2000 genes, including those of the genes associated with the plant’s immune response, hypersensitive response, phytohormone signaling pathways, photosynthesis, pigment metabolism, protein metabolism, and sugar metabolism [166]. Interestingly, both HSVd and CBCVd from cucumber and hop, respectively, altered the genes encoding for the RNA-dependent RNA polymerase, the basal defense responses as well as photosynthesis. On the other hand, comparing the hop transcripts’ data obtained from either HLVd- or CBCVd-infected hop plants revealed the high expression of four pathogenesis-related genes in hop leaves [165]. Both disease severity and the induced global transcriptome response were further studied by co-infecting hop plants with HLVd and CBCVd, which resulted in the observation of the higher expression of the genes involved in the proteolysis mechanism in the mixed infection as compared to what was seen in the single infections [154]. Similar studies on PSTVd infected tomato plants revealed a strong up-regulation of the genes involved in the plant immune responses, especially in those in the calcium-dependent protein kinase and mitogen-activated protein kinase signaling cascades, as compared to that seen in uninfected plants [152]. Viroids also accumulate in the roots, organs that are crucial for water and nutrient absorption, storage and for anchoring the plant to the ground. Within the rhizosphere, the roots are exposed to a vast and diverse microorganism community, some of which are beneficial and some of which are pathogenic. Although the majority of the studies were focused primarily on the leaves, recently an RNA-seq study has been performed on RNA extracted from the roots of PSTVd infected plants at three-time intervals post infection. The results demonstrated a differential expression of cell-wall-related genes [167], indicating that viroid infection triggers the host’s immune response irrespective of the plant tissues used for the analysis.

### 4.8. Detection and Discovery of Viroids

Due to the non-coding nature of viroid RNA, the standard serological based virus diagnostic techniques are not possible. Hence, the conventional detection of viroids was accomplished by a combination of biological indexing (i.e., bioassays) and molecular biology techniques as well as by plant certification and quarantine programs [168,169,170]. In the 1980s, different types of polyacrylamide gel electrophoreses (e.g., 2D-PAGE and reverse PAGE) and RNA gel blot assays were used for routine diagnosis [170]. These techniques were later replaced by the introduction of RT-PCR combined with the cloning and sequencing of the amplified viroid cDNAs using first-generation sequencing machines due to their precision, sensitivity, reliability, and speed [168]. Since the first RT-PCR based detection of viroids in both apple and pear trees in the 1990s [171], RT-PCR, combined with the Sanger sequencing method, has played a crucial role in the detection of a previously known viroids, or of their variants, in a new host plant or geographical area. For example, the presence of previously known viroids has been detected in new host plants such as *Brugmansia*, *Solanum jasminoides*, Dahlia (*Dahlia x hybrida*), cape gooseberry (*Physalis peruviana*), and *Lycianthes rantonnetii* [172,173,174,175,176,177]. Moreover, the distribution of viroids in new geographical areas was detected in either symptomatic or asymptomatic conditions, as described elsewhere [178,179,180,181,182,183]. NGS has also been used to detect previously identified viroids in either known host plants, or in new host plants as described previously [184,185,186]

Due to the polyphyletic genome sequence of viroids, the discovery of new viroid species using first-generation sequencing machines was not simple. The introduction of NGS has helped in both the detection of previously identified viroids, and in the discovery of new viroids as it enables total pathogen characterization without any prior knowledge of the pathogen [187,188]. As NGS permits the screening of all pathogens, it is being increasingly used in plant virology for the quick identification of both viruses and viroids via the sequencing of either total RNA or of that of short interfering RNAs (siRNA) [189]. In 2009, NGS was used for the discovery, detection and identification of viroid species. For example, the analysis of the total plant RNA sequences obtained from Syrah grapevines using the Life Sciences 454 high-throughput platform revealed the presence of a sequences of HSVd, grapevine yellow speckle viroid (GYSVd) and australian grapevine viroid (AGVd) [190]. In this approach the presence of known viroids in the sample can be proved by searching for sequence-homology using platforms such as VirFind, VirusDetect, or Virtool [191,192,193]. However, in order to detect novel viroid-like circular RNAs, either the computer algorithm has to look for a longer-than-unit length sequence, or the obtained sequence has to be circularized based on overlapping sequences prior to sequence-homology analysis using the aforementioned tools. In both cases, confirmation of the circularity of a novel RNA species has to be verified by either bioassay or PAGE analysis. Conversely, the progressive filtering of overlapping small RNAs (PFOR), a computational algorithm for the homology-independent discovery of replicating circular RNAs, applied to the total sRNAs of infected grapevine plants led to the discovery of a viroid-like circular RNA which was named the grapevine hammerhead viroid-like RNA (GHV viroid-like RNA) [194]. The application of algorithm PFOR2, an improved version of PFOR, on the sRNAs of grapevine samples revealed the presence of a new viroid, the grapevine latent viroid (GLVd) [3].

## 5. Third Generation Sequencing and Future Perspective

The first Single Molecules Sequencing (SMS) technology was developed in 2003 by Helicos BioSciences [195]. In SMS, the surface tethered poly-T oligomers are hybridized to a poly-A tailed library of randomly fragmented DNA molecules, resulting in an array of a primer annealed single-molecule DNA templates. DNA polymerase then adds a single fluorophore attached nucleotide at a time. The incorporated nucleotide is then identified with a charge-coupled device (CCD) by exciting the fluorophores with the appropriate lasers. Once the incorporated nucleotide has been identified, it is cleaved, and then the system cycles to the next base in line [196,197]. SMS avoided all of the PCR associated biases and errors as it used non-amplified DNA as the template for sequencing. The method was slow, expensive and produced only 35-nt long read lengths [198]. Hence, it was soon replaced by Single-Molecule Real-Time Sequencing (SMRT, or long-read sequencing), which was capable of producing long reads of up to 10 kb in length that made it very useful for de novo genome analysis [198,199]. Pacific Biosciences first commercialized the SMRT platform in which the DNA library was constructed by circularizing the DNA molecule to be sequenced by ligating an adaptor molecule to both ends of the target. The resulting circular DNA was then sequenced in zepto-liter (10^−21^ L) wells containing a single immobilized DNA polymerase in the presence of fluorescently labeled nucleotides [200,201]. Recent circular consensus sequencing (CCS) based approach to SMRT has generated 99.8% accurate reads with average sequence length of 13.4 kilobases (kb) [202].

Nanopore sequencing permits both the detection and the quantification of both RNA and DNA molecules [203]. The potential of nanopores for sequencing both RNA and DNA had been discussed/demonstrated in various forms prior to the development of the second-generation sequencing technologies [204,205]. The first commercial sequencer was released in 2014 by MinION (Oxford Nanopore Technology). Nanopore sequencing is based on measuring changes in electrical current as biomolecules such as DNA traverse the pore, and then using those electrical changes to identify the exact DNA base going through the pore [206]. Despite its low cost, real-time nature, and portability, the MinION platform is particularly attractive for pathogen surveillance and diagnostics, it requires reading of multiple molecules to compensate sequencing inaccuracies [207,208,209].

Third generation sequencers, especially the one that is devoid of library is very interesting as it not only reduces the error rate, but it also indicates the quantity of the target gene or nucleotide sequence at that precise moment. This kind of approach for the RNA-seq in viroid infected plants is very helpful. Currently, NGS has yielded significant insights into viroid quasi-species. However, the reconstruction of viroid molecules using vd-sRNA reads, or the preparation of the libraries using viroid specific primers, fails to produce the whole genome sequence of the viroid RNA. For example, primer based NGS libraries will not allow analysis of sequence variation in primer binding regions for quasi-species analyses. Since third generation platforms are devoid of amplification, the purified linear viroid RNA (i.e., the replicating intermediate) can be directly sequenced, or the mature circular RNA can be opened at a specific region using an RNase enzyme which facilitates the complete genome sequence of a given viroid molecule. These primer free nucleotide sequences help not only in discovering novel viroid species, but also in understanding viroid–host interactions. The development of sequencers has greatly impacted our understanding of viroid biology. It will be even more exciting to see future development in viroid research with the use of error-free sequencing technologies.

## Figures and Tables

**Table 1:** Developments in sequencing technologies and their impact on viroid research.

Year	Milestones	
	Sequencing Technology	Viroid Biology
**1960s**	RNA fingerprinting	- Chemical characterization of the first viroid
**1970s**	Detection of radiolabelled partial-digestion fragments by two-dimensional fractionation	- Sequencing of the first viroid RNA and secondary structure elucidation
**1980s**	Sanger’s chain-termination technique Maxam and Gilbert’s chemical degradation technique	- Determination of the structural/functional domains, central conserved region (CCR), and hammerhead self-cleavage - Viroid classification - Viroid isolates
**1990s–to date**	Automated sequencers	- Mutagenic studies - Structure of viroids - Quasi-species
**2005–to date**	Next-generation sequencers	- Characterization of viroid derived small RNAs (vd-sRNA) - Quasi-species: Large scale mutation rates and population dynamics - Effect of viroid infection host genome: Transcriptome analysis and degradation studies
